# Asexual Evolution and Forest Conditions Drive Genetic Parallelism in *Phytophthora ramorum*

**DOI:** 10.3390/microorganisms8060940

**Published:** 2020-06-22

**Authors:** Jennifer David Yuzon, Renaud Travadon, Mathu Malar C, Sucheta Tripathy, Nathan Rank, Heather K. Mehl, David M. Rizzo, Richard Cobb, Corinn Small, Tiffany Tang, Haley E. McCown, Matteo Garbelotto, Takao Kasuga

**Affiliations:** 1 Department of Plant Pathology, University of California, Davis, CA 95616, USA; rtravadon@ucdavis.edu (R.T.); hkmehl@gmail.com (H.K.M.); dmrizzo@ucdavis.edu (D.M.R.); cssmall@ucdavis.edu (C.S.); tsytang@ucdavis.edu (T.T.); hemccown@ucdavis.edu (H.E.M.); 2 CSIR Indian Institute of Chemical Biology, Kolkata 700032, India; mmadhubioinfo@gmail.com (M.M.C.); tsucheta@gmail.com (S.T.); 3 Department of Biology, Sonoma State University, Rohnert Park, CA 94928, USA; rank@sonoma.edu; 4 Department of Natural Resources and Environmental Science, California Polytechnic State University, San Luis Obispo, CA 93407, USA; rccobb@calpoly.edu; 5 Department of Environmental Science, Policy and Management, University of California, Berkeley, CA 94720, USA; matteog@berkeley.edu; 6 Crops Pathology and Genetics Research Unit, USDA Agricultural Research Service, Davis, CA 95616, USA

**Keywords:** asexual reproduction, parallel evolution, *Phytophthora ramorum*, Structural Variants, forest pathology

## Abstract

It is commonly assumed that asexual lineages are short-lived evolutionarily, yet many asexual organisms can generate genetic and phenotypic variation, providing an avenue for further evolution. Previous work on the asexual plant pathogen *Phytophthora ramorum* NA1 revealed considerable genetic variation in the form of Structural Variants (SVs). To better understand how SVs arise and their significance to the California NA1 population, we studied the evolutionary histories of SVs and the forest conditions associated with their emergence. Ancestral state reconstruction suggests that SVs arose by somatic mutations among multiple independent lineages, rather than by recombination. We asked if this unusual phenomenon of parallel evolution between isolated populations is transmitted to extant lineages and found that SVs persist longer in a population if their genetic background had a lower mutation load. Genetic parallelism was also found in geographically distant demes where forest conditions such as host density, solar radiation, and temperature, were similar. Parallel SVs overlap with genes involved in pathogenicity such as RXLRs and have the potential to change the course of an epidemic. By combining genomics and environmental data, we identified an unexpected pattern of repeated evolution in an asexual population and identified environmental factors potentially driving this phenomenon.

## 1. Introduction

Asexuality in eukaryotes presents a challenge to the diversification of genotypes, yet many asexual eukaryotic lineages persist, showing considerable genetic diversity, and have the ability to adapt to novel and changing environments [1,2,3,4,5,6,7]. Genetic variation in asexual organisms arises from various modes such as rare sexual recombination or cryptic meiosis [8], horizontal gene transfer (HGT) [9], and somatic mutations [6]. In the case of somatic mutations, large scale somatic mutations known as Structural Variants (SVs) can create alterations to the contents of the genome, which may lead to major effects on the phenotype [10,11].

*Phytophthora ramorum* (Stramenopiles Peronosporales), an oomycete plant pathogen, is an asexually reproducing organism able to colonize and persist in new ecosystems. The pathogen consists of four known, asexually propagating lineages introduced to the United States and Europe. The *Phytophthora ramorum* NA1 lineage, the causal agent of Sudden Oak Death (SOD), found in California has devastated natural forest lands since the mid-1990s [12,13,14]. The pathogen has a heterothallic mating system likely derived from a sexual population [15,16,17]. However, only a single mating type, A2, has been identified in California forests and its sexual reproduction has never been observed in nature [18]. Instead, asexual propagules produced during warm and spring rain conditions occur each year, accounting for the vast majority of infections locally and at landscape scales [19]. Previous studies of the California NA1 lineage employing microsatellite markers have shown higher than expected observed heterozygosity under the Hardy–Weinberg equilibrium and have revealed an accumulation of identical multilocus genotypes (MLGs). These findings are consistent with asexual reproduction and the absence of recombination [20].

Though *P. ramorum* NA1 propagates asexually, associations with distinct plant species have been connected with alterations in genomic regions, gene expression, and phenotypic traits of *P. ramorum* isolates [11,21,22]. The pathogen has a wide host range and infects over 100 species of plants [23], both wild and ornamental (e.g., *Quercus* spp., *Umbellularia californica*, *Sequoia sempervirens*, *Rhododendron* spp., and *Camellia* spp.), causing leaf lesions and/or branch dieback [12,19,23,24,25]. During its infectious stage in the trunks of the non-transmissive *Quercus spp* hosts, *P. ramorum* is known to permanently form chromosomal aberrations and phenotypic changes [10]. Upon closer inspection of the genetic mutations involved, it was shown that the same haplotypes increased in copy number between isolates from different hosts. This phenomenon was termed Host-Induced Phenotypic Diversification (HIPD) and was presumed to occur when the pathogen interacts with non-transmissive hosts such as species of *Quercus* [21]. Structural Variants were shown to occur in *P. ramorum* cultured from *Rhododendron* spp. and other non-*Quercus* hosts, albeit at a lesser extent, suggesting that a similar phenomenon may occur when *P. ramorum* associates with “transmissive hosts”. Alternatively, the possible use of fungicides in ornamentals could be in part responsible for the generation of Chromosomal Copy Number Variation (CCNV; a type of SV) [11]. In addition to CCNVs, there are other classes of SVs such as deletions, amplifications, inversions, and translocations that are ubiquitous in the *P. ramorum* genome, and are only detectable by genome sequencing. Such mutations are common in the genus *Phytophthora* and have also been identified in *P. infestans* [26], *P. capsici* [27], and *P. cinnamomi* [28]. How SVs are generated in transmissive hosts and whether these mutations are transmitted to future generation of *P. ramorum* NA1 has yet to be determined.

Extensive ecological studies have identified biotic and abiotic factors in the forest that influence the demography (e.g., survival and pathogen spread) of *P. ramorum* NA1. The distribution of hosts in forest ecosystems are known to drive population size expansion and pathogen survival. On the main transmissive hosts such as California bay laurel (*U. californica*) and tanoak (*Notholithocarpus densiflorus*), the pathogen produces infectious spores responsible for cross-scale spread and disease intensification [29,30]. During the summer, the survival of the pathogen also depends on host density and forest composition [31,32]. Hence, *U. californica* and *N. densiflorus* are considered the most epidemiologically relevant host for *P. ramorum* and collectively the abundance of both species has the greatest predictive capacity for forecasting spread and disease emergence [12,19,24,31,33]. When precipitation increases, especially during the spring, sporangia form on infected leaves and release zoospores that are transmitted via rain splash and wind [31,32]. Though abundant during spring, survival is reduced by higher temperatures, and correlated with canopy cover (a proxy for sun exposure) [31,32]. Because the pathogen population is affected by host density, temperature, solar radiation and precipitation, these environmental variables may also be involved in the generation of new genetic material in *P. ramorum* NA1 and their contribution to extant lineages.

We explore the molecular, evolutionary, and environmental conditions associated with the emergence of parallel or functionally similar SVs across different populations of asexual *P. ramorum* NA1. We hypothesized that SVs were either generated somatically or by recombination between individuals. Second, we asked if SVs are transmitted to extant lineages or were evolutionarily short lived. Third, we asked if epidemiologically relevant factors are likely driving the generation of parallel SVs. We tested a set of environmental variables known to be involved in the survival or spread of the pathogen. Parallel SVs could have the potential to affect the biology of the pathogen as they repeatedly arise in genes coding for pathogen effectors and genes that are upregulated during in planta interactions with *N. densiflorus*. Our findings not only reveal how genetic variation in an asexual organism is generated at the molecular and ecology levels, but also suggest that an underlying process other than chance drives genetic diversity in regions of the genome related to pathogenesis. Therefore, our results may provide the basis for future studies of adaptation in the asexual *P. ramorum* to its new forest environment and forecasting of future outbreaks.

## 2. Materials and Methods

### 2.1. Isolates and Phenotyping

**Figure 1:**
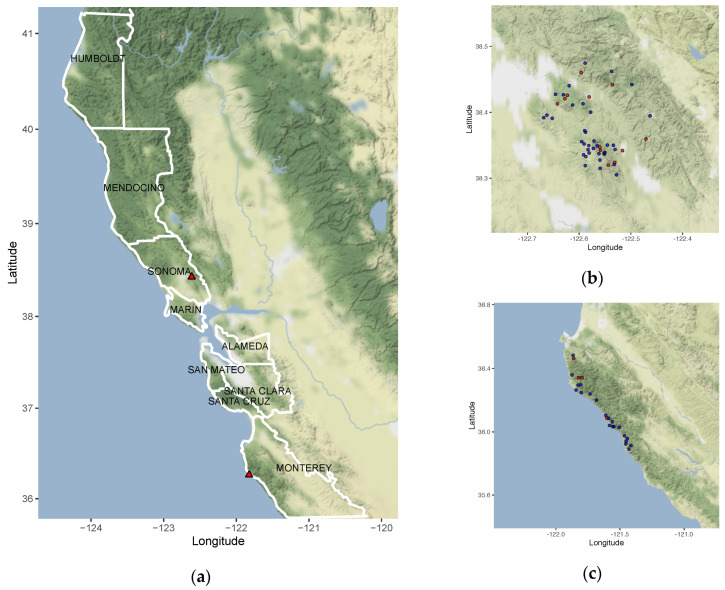
Maps of California counties where *P. ramorum* isolates were collected. Longitude and latitude are shown on the x- and y-axis. (**a**) Left panel shows the counties outlined in white where isolates used in the phylogeny (Figure 2, and Figure 4) were collected. Red triangles refer to Sonoma Co. and Monterey Co. which are zoomed in on the (**b**) top right and (**c**) bottom right panels. Red circles indicate plots where non-wild type (NWT) isolates were collected, and blue circles show where wild type (WT) isolates were collected. Isolates collected from (**b**) Sonoma Co. and (**c**) Monterey Co. plots where used in the environmental association study (Figure 6).

A total of 78 *P. ramorum* isolates from California were examined to analyze the evolution of SVs and their association with environmental conditions (Table S1). Forty-seven of the isolates were used to study population structure throughout coastal California (Figure 1). Sonoma Co. and Monterey Co. isolates with geographical coordinates were used in the genomic–environmental association analysis. Isolates were previously used in population genetic and phenotypic research [10,11,34,35,36,37,38,39].

Since the “non-wild type” (NWT) phenotype is associated with SVs [10], we focused on this character trait to identify isolates that are likely to have SVs. One hundred and two isolates from Sonoma Co. and 92 isolates from Big Sur forests of Monterey Co. (from here identified as “Monterey Co.” for clarity) were phenotyped in culture as previously described [10,11]. NWT is defined by a 25% smaller diameter compared to the average WT cultures or at least 15% deviation in radius within a 45° section of the colony. “Wild-type” (WT) colonies usually have a uniform growth pattern. Phenotyping was repeated twice and cultures were maintained and harvested as in Kasuga et al., 2016 [10].

### 2.2. DNA Extraction and Libraries

Genomic DNA extraction followed the method cited in Kasuga et al., 2016 [10]. Paired-end libraries with a 350 base pair insert size were made for each isolate according to the manufacturer’s instructions for TruSeq DNA LT Sample Prep Kit (Illumina, Inc, San Diego, United States). Collection and sequencing information for each isolate can be found in Table S1. Sequences were deposited in NCBI-SRA (accession: PRJNA558041 and PRJNA559872).

### 2.3. Microsatellite Analyses

The population structure of *P. ramorum* NA1 was first reconstructed using microsatellite markers [34,35,36] and were compared to results from the SNP phylogeny. The 42 isolates were genotyped using six microsatellite loci (Table S2), PrMS39a, PrMS39b, PrMS45, PrM43a and PrMS43b [40] and locus 64 [41] using primers and thermal cycling conditions previously described [36,40,41]. Successful PCR amplifications were verified on 1.5% agarose gels and PCR products were subsequently sized on an ABI PRISM 3130xl sequencer (Applied Biosystems, Foster City, United States) using Rox 500 as size standard. Allele size assignments were performed using Genemarker (SoftGenetics LLC, State College, United States). Allelic data were formatted for the program GenAlex6 [42] which was used to identify identical MLGs among the 42 isolates. To illustrate the genetic variation among unique MLGs, a neighbor-joining tree was constructed based on Nei’s genetic distance, Da [43] using the program POPULATIONS 1.2.30 [44]. The tree was visualized using Nei’s genetic distance to identify points of reticulation. The microsatellite network was visualized on the program SplitsTree4 [45].

### 2.4. SNP Calling

To reconstruct the genealogy of the *Phytophthora ramorum* NA1 California population, we called SNP markers using the most complete genome assembly of the pathogen. *P. ramorum* has a diploid genome with an estimate of 10–12 pairs of chromosomes (unpublished, C. Brasier, Forest Research— Forestry Commission UK). The most complete genome assembly is of isolate ND886 and has an unphased genome assembly of 302 contigs and is 60.2 MB. The size of the contigs ranges from 1.6 MB to 177 bp [38]. Reads were aligned and filtered using the protocol in Malar C et al. (2019a) [38]. Loci where at least 2/3 of ND886 samples and 3/4 of the Pr1556 samples had consensus were retained for analysis. ND886 was isolated from an ornamental host and was used for the reference genome. Pr1556 was isolated from a forest environment and was used in many SV analyses [10,11]. To eliminate SVs that could interfere with phylogenetic reconstruction, TitanCNA [46] was used to identify and mask SVs. Scripts for all the analyses shown in this paper have been deposited at https://github.com/jdyuzon/pramNA1-CApop.

The genomes of asexual organisms can have higher levels of heterozygosity than expected compared to sexual lineages [47,48]. When calling polymorphic sites, 237,057 fixed heterozygous sites interfered with the identification of SNPs potentially useful to describe *P. ramorum* population structure. To obtain phylogenetically informative SNPs, loci with a majority of homozygous sites and at least one heterozygous site were retained, assuming that the probability of a SNP to occur multiple times at the same genomic location was low. Phylogenomic reconstruction of diploid organisms can be problematic because of the ambiguity of markers in heterozygous regions. However, since *P. ramorum* NA1 is asexual and a recently established population, we assume that the SNP markers are less likely to be homoplasic. Next, we filtered out loci that were at least 50 bp within proximity to each other, which is less than the average density of fixed heterozygous SNPs (approximately every 250 to 300 bp).

Relationships based on the final SNP calls were reconstructed (Figure 1a) on SplitsTree4 using the Splits Decomposition method [45] to identify any discordances that may indicate recombination among isolates or sequencing error. In Splitstree4, if alternative relationships are present in the evolutionary history of a set of taxa, these relationships would be represented as reticulation and the phylogeny would appear as a network rather than a bifurcating tree.

### 2.5. Structural Variant Calling

To further identify which alleles are affected by SVs, the phased ND886 genome assembly [38] was used to call SVs. In the phased ND886 genome assembly [38], there are 345 haplotype blocks and 222,892 phased variants across 302 contigs (60.2 MB). The largest haplotype block is 1.5 MB with 7265 phased variants. Reads were binned to either of two FASTA files containing haplotype 1 and haplotype 2 of the phased genome assembly of ND886 [38] using BBSplit [49]. Reads were aligned to their best haplotype match using bwa aln [50] and files were then merged using SAMtools (version 1.3.1, Cambridge, United States) [51,52].

SVs were called using read depth, split-read, and paired-end methods. Copy number variants were called using the read depth method of TitanCNA [46]. SV calling using TitanCNA followed the protocol in Malar C et al. (2019a) [38]. Similarly, BAM files were analyzed using paired-end-and split-read-aware prediction methods of Delly with post-filter [53] and LUMPY [54].

For the final SV set, SV calls between the different callers were compared. To confirm SVs within an isolate, SV calls from TitanCNA were filtered for regions with at least four supporting heterozygous sites. SV intervals were merged based on the average distance separating intervals. We observed that large SVs seen in the TitanCNA graphs were comprised of disjointed intervals in the text files. Therefore, we assumed that intervals that were close to each other within an isolate were actually from a larger SV. For all SV callers, regions that had both amplification and deletion calls with at least 0.5 fraction overlapping intervals were removed from the dataset. We assumed that amplifications and deletions could not possibly occur at the same site within the same isolate. However, we also assumed that one isolate could have complex mutations of amplification or deletion, and translocation, inversion or insertions. Therefore, amplifications and deletions that overlapped with translocations, inversions, or insertions were retained and are represented in our inferences. ND886 and Pr1556 were controls for SV analysis, therefore SVs called by TitanCNA, Delly and/or Lumpy that overlapped with SVs called in ND886 and Pr1556 were filtered and removed. Since isolate Pr102 is associated with a non-transmissive host [12], which could introduce bias, normal states were assumed for this isolate. All SVs from TitanCNA were kept, whereas Lumpy and Delly calls were only kept if they overlapped with one other caller. SV calls for isolates were compared at the population level and SVs that had a 0.8-fraction overlap were assumed to be in the same locus. SV loci that had at least two SVs, even if they belonged to different types (e.g., amplification and translocation or amplification and deletion) were retained.

### 2.6. Test of Asexual Evolution

Somatic mutations and recombination between individuals were assessed to understand whether *P. ramorum* can generate genetic variation by occasional sexual recombination in a predominantly asexual system. First, recombination between individuals was tested to detect meiotic recombination. Linkage disequilibrium was summarized with the r^2^ statistic in PLINK [55] for all 716 SNPs. Second, phylogenetic reconstruction using the Splits Decomposition method on Splitstree4 [45] was used to identify any major discordances in the unphased and phased phylogeny. Third, the phylogeny of each phased contig was reconstructed using phased SVs with heterozygous sites. If haplotypes A and B of isolates were mixed with each other, this would suggest recombination. If haplotypes A and B formed separate and distinct clades, this would be a signature of an asexual population. If the alleles clustered together by isolate in a phylogeny, this would suggest somatic mutation by gene conversion.

### 2.7. Phylogenetic Reconstruction and Ancestral State Reconstruction

The shape of a tree can be influenced by character traits if these traits are involved in selection which can be mediated by estimating a character’s state on the tree’s branching process [56]. A joint model of tree reconstruction and trait change was employed to estimate the tree and trait dwelling times and transitions between states. We used the Birth–Death Serial Sampled (BDSS) model [57] with tips dated by year (Table S1). The alignment consists of the same SNPs of the phylogenetic network that was represented as two unphased nucleotides. To represent invariant sites, we set constant weights for A, C, G, and T (27421982, 32889740, 32791456, and 27520622), which were not represented in the original alignment. The SNP and SV branch-rate priors were under an uncorrelated lognormal clock, and we used the GTR SNP substitution model. Using bModelTest [58], an unnamed substitution model nested in the GTR model was identified as the best match for our dataset. We used an asymmetric model for SV substitution in which the following rates were set to zero: deletion to normal, translocation to amplification, and deletion to amplification. The root was weighted by a fixed vector assuming that it started at a normal state. Six independent runs were performed on BEAST v1.8.0 [59] with BEAGLE 3.0.2 [60] for 20,000,000 generations, sampling every 10,000 iterations.

To assess how long an SV persisted in the population, we implemented fast stochastic mapping, which estimates dwelling times for the population [61,62]. We therefore refer to dwelling times as “persistence times”. The first 10% of trees were discarded. All SV persistence times and mutation counts chains converged with high Effective Sample Size (ESS, independent samples from the posterior distribution) that rarely fell below 200 and never below 100. From the combined log files, mean persistence times and mutations counts were calculated for each SV. For example, persistence times of amplifications were longer than translocation and deletion persistence times. SV loci were clustered in a K-means analysis based on persistence times and mutation counts. Optimal number of clusters were calculated using the Silhouette Method using R packages factoextra and cluster [63,64]. Isolates with SVs were also grouped using the same clustering method for SV loci.

Trees for independent runs were combined using logcombiner with the first 10% of trees removed followed by a resampling of every 50,000 trees, which were summarized using common ancestor heights on TreeAnnotator 1.8.0. With the fixed tree, the complete SV history on all branches [65] was estimated. The SV branch-rate prior and substitution model are the same as before with the root assumed as beginning at the normal state. The analysis was run for 10,000,000 generations. The first 20% of trees were removed with resampling every 50,000th iteration. Only 160 trees were generated from a single run on BEAST v1.8.0 because of a computational burden in summarizing per branch persistence times and mutation counts using custom scripts. Trees files from the complete history analysis were post-processed using scripts generously provided by Jiansi Gao, UC Davis. These scripts generated 158 phylogenies for 158 SV. From these trees, we were able to count the number of parallel, transmissive, and parallel–transmissive SVs.

From the full history, each branch was tested for greater persistence times compared to all branches in the phylogeny. To create the null distribution for each SV, average persistence times were calculated from the posterior distribution of the population of all branches. Some branches did not have a persistence time for certain SV types (e.g., no deletion persistence times because the observed SV is an amplification) or no transition (e.g., transition occurred earlier in the history on a prior branch), therefore all missing data and infinite values were removed. A Mann–Whitney U-test was performed for each branch in comparison to the average persistence time of all branches and to calculate the *p*-value. All SVs within a branch with a *p*-value less than 0.05 were counted after Bonferroni correction. Custom genomic analyses scripts can be found in the GitHub repository: https://github.com/jdyuzon/pramNA1-CApop.

### 2.8. Environmental Sampling and Modelling

To examine the association of phenotype and genotype with environmental conditions, environmental data were compared to NWT phenotype and SV content. Of the 78 isolates, only 22 isolates from Monterey Co. and 27 isolates from Sonoma Co. had full climate and forest structure data associated with their plots. These climate data were collected from BioClim [66] and PRISM from the 30 year normal which is approximately the duration of the epidemic. Stem density, and elevation at the plot level (500 m2) were collected in the field. To test if NWT/WT phenotypes can be explained by environmental conditions, a generalized linear model with a binomial distribution and logit link was constructed. The independent variable were pairwise comparisons of environmental conditions associated with isolates from Monterey Co. and Sonoma Co. Similarity in environmental conditions between demes were represented as a Jaccard’s distance using the package philentropy [67] in R statistical software. A random effect was included for isolate identity. We also investigated the relationships between NWT/WT phenotype and SV content. A generalized linear model with a binomial distribution and logit link was used to test for phenotype in response to the number of parallel SVs, total SVs, amplifications, translocations, and deletions. SV types were highly correlated, so separate models were constructed. To know if environmental conditions predict the number of parallel mutations, we again calculated the Jaccard’s distance between all pairwise comparisons of environmental variables associated with isolates from Monterey Co. and Sonoma Co. Similarity in environmental conditions was used in generalized linear models (with a hurdle Poisson distribution and log link) as independent variables with the number of parallel SVs between pairs of isolates as the response variables. Isolate identity was included as a random effect. Model adequacy and convergence are shown in plots comparing the observed response variable “y” to simulated datasets “y_rep_” (posterior predictive checks) and trace plots.

### 2.9. Association with Genomic Features

To determine genes and repetitive regions that may be affected by SVs (effectors, repetitive elements, and other genes identified using the ND886 transcriptome in culture), a Fisher’s Exact test was used to establish whether the number of overlapping base pairs between SVs and genomic features were less than or greater than expected, given the number of SV base pairs that overlap with the genomic feature. The SVs, genomic features, and reference genome included both haplotypes. The in planta transcriptome is of MK1461 (isolated from *U. californica* found in San Mateo Co.) inoculated onto *N. densiflorus* (unpublished, M. Garbelotto, Forest Pathology and Mycology Lab— UC Berkeley) and was used as a reference for the Fisher’s Exact test.

To assess whether the identified SVs had biological relevance for the pathogen, genic regions excluding repetitive elements and effectors were subjected to Gene Ontology (GO) enrichment and compared to the genome. First, genes were annotated using InterProScan (version 5.31-70.0, Cambridge, United Kingdom) [68]. GO enrichment was performed using GOstats [69] with the annotated genome as background, and *p*-values were adjusted using a false discover rate [70].

A Fisher’s exact test was used to determine if SV mutation counts and persistence times were associated with isolate host or substrate. To construct the contingency table, isolates were identified as originating from common or uncommon hosts and sources. The second grouping was based on the K-means analysis of SV counts and persistence times.

## 3. Results

### 3.1. SNP Markers Confidently Reconstruct the Phylogeny of P. ramorum NA1

**Figure 2:**
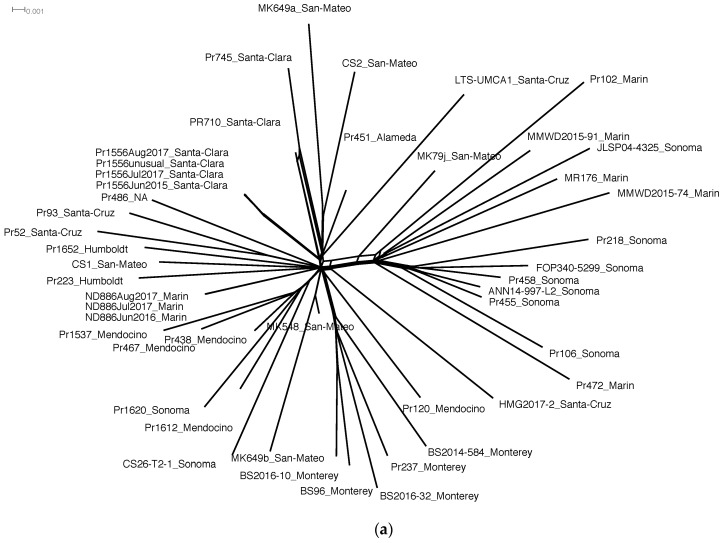

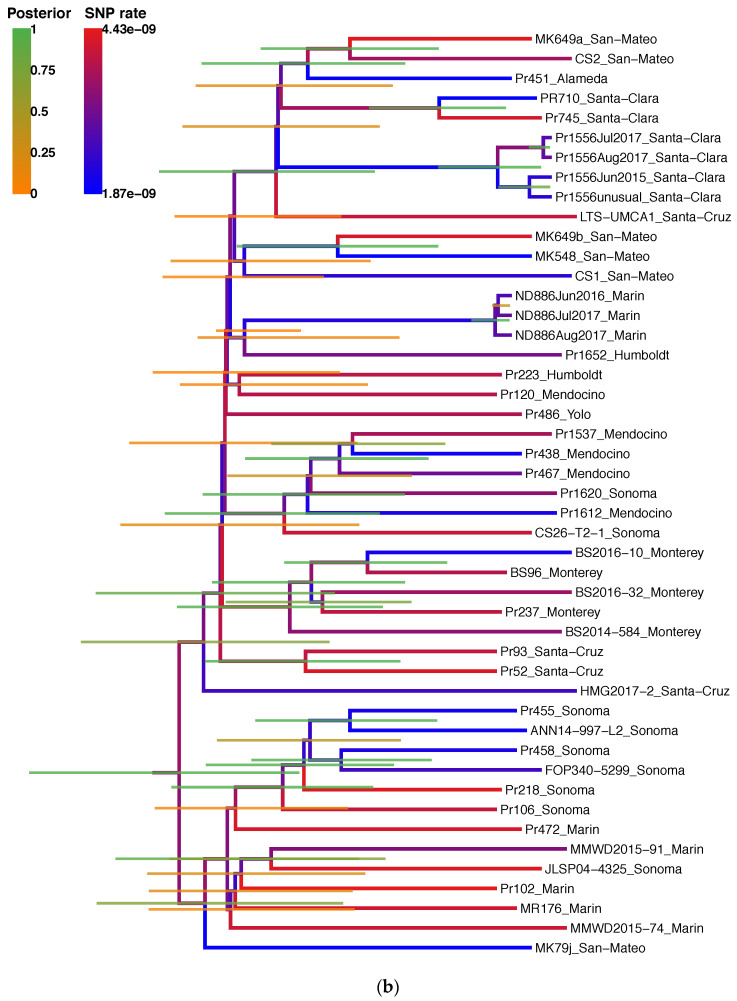
Phylogenetic reconstruction of *P. ramorum* NA1 in California. (**a**) Splitstree graph using SNPs indicates resolved relationships and discordances as links between branches; (**b**) phylogeny using BEAST v1.8.0 under the Birth–Death serial sample model. Node labels are the posterior probabilities of node height and line color represent posterior support of SNP mutation rate. Isolate identification numbers are followed by the California county of origin and naming conventions are consistent with previous publications. The first letters of the identification number represent the original project or the collector (e.g., BS = Big Sur project, and MMWD = Marin Municipal Water District). Pr1556 and ND886 served as controls during sequencing and the dates following their name indicate the time they were sequenced (e.g., August 2017).

In order to determine the mode by which SVs develop in parallel evolution, accurate reconstruction of genetic relationships is required between demes as well as between individuals. To this end, we mapped reads of 42 isolates (Table S1) to the unphased ND886 genome, leading to the detection of only 716 polymorphic SNPs out of the 237,773 heterozygous SNPs called in the *P. ramorum* population. Therefore, 237,057 loci (99.7%) were fixed heterozygous loci, representing phylogenetically uninformative heterozygosity in the ancestral strain. In total, 716 polymorphic heterozygous SNPs were used to reconstruct the phylogeny (Figure 2a,b). Read coverage ranged from 30X to 145X when aligned to the unphased genome (Table S1). Eleven SNPs distinguished the closely related isolates Pr710 and Pr745 from each other. While phylogenetic analysis showed 85 synapomorphic SNPs, these results successfully confirmed previous studies of the California *P. ramorum* NA1 population by Croucher et al., 2013 [36], and Mascheretti et al., 2008 [34] and 2009 [35]. Specifically, in the earliest diverging clade of the two major clades, five isolates were from Marin Co., Sonoma Co. (7), and Santa Cruz Co. (3). Isolates from the Big Sur region in Monterey Co. formed a clade separate from the rest of the population (Figure 2b). Though the phylogeny has only 716 markers with 85 shared SNPs, our ancestral state reconstruction methods, as with other Bayesian inference methods, take into account uncertainty, thus providing robust distinctions between isolates for the reliable identification of genetic relationships.

In order to confirm that taxon groupings were non-erroneous and that no interference in evolutionary signal could be attributed to technical errors, we examined conflicts in the SNP phylogeny using a phylogenetic network approach. Genetic homoplasy can confound phylogenetic reconstruction because shared mutations that arose multiple times may lead to erroneous taxon groupings. In addition, high-throughput sequencing is subject to technical errors and can interfere with evolutionary signals. To determine if the SNP markers used to reconstruct the genealogy of the population had limited homoplasy and to check for technical errors, we used the splits network representation to visualize any discordance in the phylogeny. In total, 97.8% of branches were resolved and replicate sequences of controls ND886 and Pr1556 formed closely related groups (Figure 2a, b).

In order to generate a phylogenetic reconstruction for estimation of SNP mutation rates from the time calibration of the serial sampling, we employed a Bayesian inference method (Figure 2b). This phylogenetic analysis revealed an estimated mutation rate of 1.87 to 4.44 × 10^−9^ mutations per bp per year, close to mutation rates of eukaryotes, which ranges from 10^−8^ to 10^−9^ [71,72,73,74,75]. Since our samples have known collection dates (Table S1), the trees estimated by BEAST v1.8.0 were time calibrated and automatically rooted.

Next, we checked for sequencing errors or SNP accumulation during culture that can potentially hide true genetic relationships in the phylogenetic tree by estimating technical and biological error in the ND886 and Pr1556 controls. Among the three sequencings of ND886, June 2016, July 2017, and August 2017, we found no nucleotide variation except at two positions with missing information. For Pr1556, there were three SNP differences that separated 2015 and 2017 sequencing results. The three SNPs were heterozygous and showed the same nucleotide substitutions between July and August 2017, indicating that mutations generating these SNPs likely occurred during strain culture.

We then compared the phylogenetic network (Figure 2a) to the microsatellite tree (Figure S1), since microsatellite markers were the only genetic markers used to date to characterize the population structure of *P. ramorum* NA1. Among the 42 isolates analyzed, 33 MLGs were obtained by microsatellite genotyping. One MLG was shared by five isolates (Pr120, Pr451, Pr486, MK649a and Pr1556), one MLG was shared by three isolates (HMG2017, Pr237 and Pr1612), and three MLGs corresponded to three pairs of isolates (MK649b and MK548; BS96 and Pr438; JLSP04-43 and Pr93). The microsatellite network tree (Figure S1), constructed from the allelic variation at six microsatellite loci (Table S2), showed a high level of genotypic similarities (31.9%) and the phylogenetic structure was difficult to discern compared to the SNP phylogeny (Figure 2a,b) and previously published results [34,35,36]. Given the small sample size and number of markers, the microsatellite data could not reconstruct relationships between isolates in the California population.

### 3.2. SNPs and SVs Support Asexual Evolution

Multiple mechanisms, such as somatic mutation and recombination (HGT and meiosis), can give rise to genetic variation spanning multiple base pairs such as SVs. To establish the mode of reproduction in *P. ramorum*, we first inspected the 716 SNP markers used for the population genealogy. We tested whether there was recombination indicating intraspecies genetic exchange between individuals by using r^2^ as a statistic for linkage disequilibrium. For the unphased dataset, r^2^ on average equaled 1.0 across the whole genome, including all loci with SNPs, which is consistent with asexual reproduction. Asexual reproduction was further supported by minimal discordance in the SNP genealogy of *P. ramorum* NA1. Only polymorphic SNPs (716) were used for phylogenetic network analysis on Splitstree4 (Figure 2a) and further phylogenetic analyses (Figure 2b, Figure S2). The SNP dataset showed minimal discordances except near main branches connecting all isolates, thus indicating that the SNP markers supported an asexual mode of reproduction.

**Figure 3:**
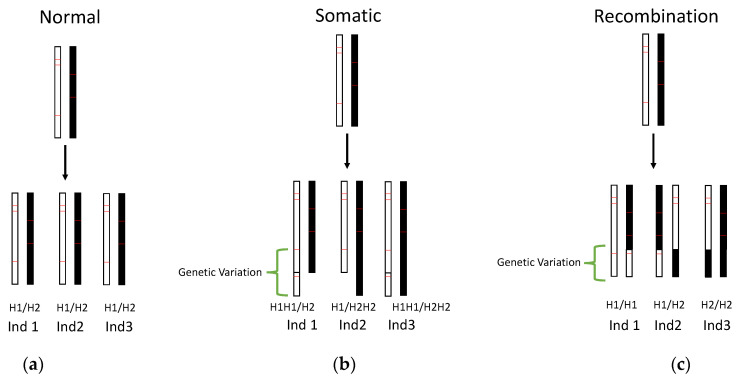
Processes that lead to genetic variants include somatic mutations and recombination. Three hypothetical scenarios are shown: (**a**) Normal state [no Structural Variants (SVs)]; (**b**) SV arising from somatic mutation; and (**c**) genetic variation (arising from recombination between individuals). Three individuals are represented (e.g., Ind 1, 2, and 3) and their corresponding haplotypes (H1 and H2). Haplotypes: H1, black; H2, white. Green brackets, genomic regions with variation; red lines, hypothetical SNPs that distinguish haplotypes. (**b**) Individuals with somatic SVs (e.g., amplification, assuming duplication occurs by tandem duplication) show an increase in haplotype (H1H1/H2, H1/H2H2, or H1H1/H2H2). (**c**) Genetic variants from recombination are likely to have allele combinations representing genotypes found in a Hardy–Weinberg equation (i.e., H1/H1, H1/H2, and H2/H2).

We next sought to compare the phylogeny of genetic variants with that of population genealogy, since congruence between phylogenies would support somatic mutation as the source of genetic variation while incongruence would support genetic variation caused by recombination. However, insufficient SNPs were identified in the genetic variants to permit fine scale phylogenetic analysis necessary to identify the mechanism generating new mutations. To address this issue, we instead compared the location of these SV mutations on haplotypes. This analysis revealed 158 regions with 140 amplifications, 13 translocations, and 5 loci with a combination of amplifications and translocations or deletions. Further analysis of the haplotype relationships (H1, haplotype 1; H2, haplotype 2) of these SVs did not show any allele combination in a Hardy–Weinberg equilibrium equation rejecting recombination (e.g., H1H1, H1H2, and H2H2; Figure 3). Instead, allele combinations showed H1/H2 change to H1/H2H2 (H2 amplified, Supplementary File S1). Occasionally (12/158 genetic variants), we saw both alleles amplified (e.g., H1/H2H2, H1H1/H2, H1H1/H2H2 (H1 and H2 amplified)). In the twelve cases where both haplotypes were affected by SVs, SVs occurred in isolates (e.g., Pr455 and BS2014-584) with elevated mutation counts (median values 24.14 and 0.15 for high and low mutation counts, respectively; Mann–Whitney U test *p*-value = 8.6 × 10^−4^). Together, the SNP phylogeny on Splitstree, high Linkage Disequilibrium, and the absence of Hardy–Weinberg equilibrium support somatic mutations as the mechanism generating genetic variation.

### 3.3. Persistence of SVs in the Population is Associated with Host and Mutation Load

**Figure 4:**
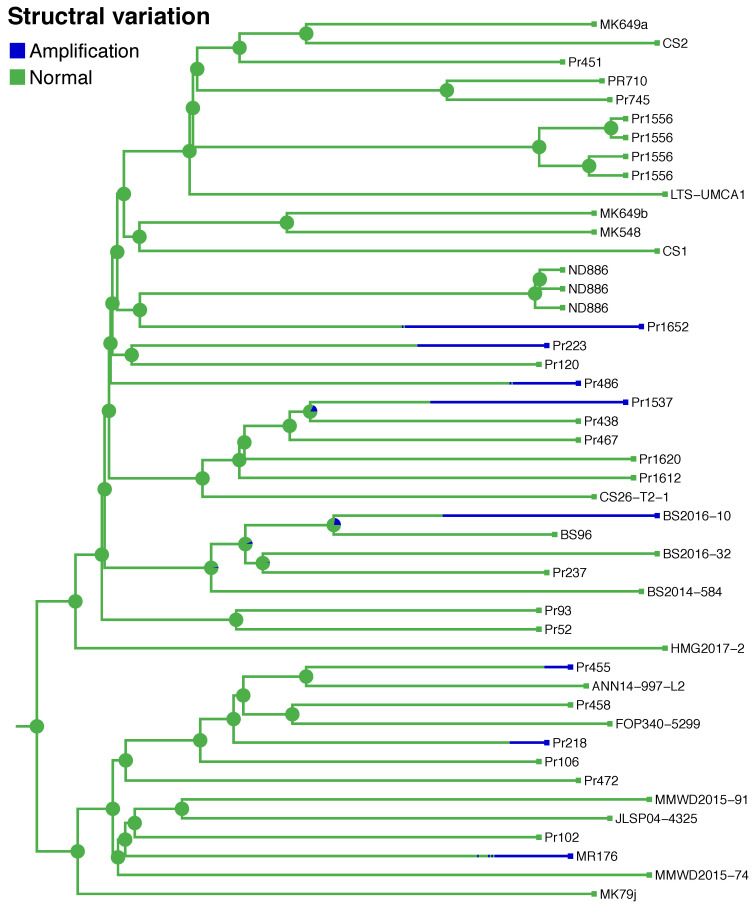
Ancestral state reconstruction of SVs indicates patterns of parallelism and estimates of persistence time. Example phylogeny testing duration of persistence time comes from SV117. The legend in the top left corner indicates the SV type (blue for amplifications, and green for normal state). The branch lengths correspond to time in years. Branches leading to Pr1652, Pr1537, and BS2016-10 had longer persistence times in the amplification state compared to branches leading to Pr223, Pr486, Pr455, Pr218, and MR176. Pie charts at each internal node indicate the posterior estimate of the SV ancestral state.

In order to estimate the frequency of transmission of SVs to future generations of the population, the persistence times (the length of time an evolutionary lineage spends in a particular trait state), and the number of times each SV independently arose in the tree were calculated using ancestral state estimation. From the stochastic mapping analysis in BEAST v1.8.0, the genome-wide mutation rate for SVs varied from 2.5 × 10^−4^ to 8.9 SVs per year. The duration of persistence times for each branch (represented by the length of the blue lines) were the average values taken from the posterior distribution of 100 trees (Figure 4). For example, the subtending branch of BS2016-10 had a longer average dwelling time in comparisons of SV persistence times in the posterior distribution to all other branches and their 100 simulated persistence times. A Mann–Whitney U-test was performed between the two distributions of the test branch and the population branches. Branches leading to isolates Pr1537 and Pr1652 also had longer persistence times in the amplified state than other branches (Figure 2).

**Figure 5:**
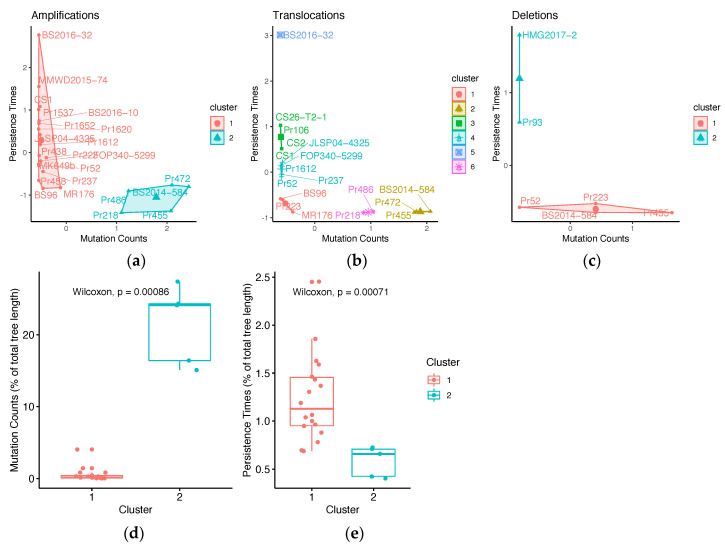
SV persistence times and mutation counts of isolates. K-means clustering grouped isolates with SVs into (**a**) two groups of amplifications, (**b**) six groups of translocations, and (**c**) two groups of deletions. Each point represents an isolate with their corresponding persistence time (scaled by branch length) vs. mutation count. Numbers of clusters were determined by the Silhouette Method. Significant differences in mutation counts (**d**) and persistence times (**e**) were examined for the two clusters in the K-means analysis of amplifications. The two clusters of amplifications represent isolates with higher persistence times (1.1%) and lower mutation counts (0.2, Cluster 1), and isolates with lower persistence times (0.7%) and higher mutation counts (24.1, Cluster 2).

Previous research showed that the non-transmissive host *Quercus* causes genomic aberrations in *P. ramorum* [10]. To determine if isolate host association is correlated with SV count and persistence times, we performed Fisher’s Exact test. First, isolates were categorized by their collection from either common hosts and sources, or from uncommon hosts and host tissue (Table S1). Common substrates are defined as hosts and substrates that the pathogen frequently associates with such as *Umbellularia californica*, *Notholithocarpus densiflorus* twigs, *Rhododendron, Camellia*, and stream water. Uncommon sources are hosts and substrates that the pathogen can infect but are not the main sources of transmission (e.g., *Osmorhiza berteroi*, *Frangula californica*, *Choisya ternata*, *N. densiflorus* bark). Second, a K-means clustering analysis (Figure 5a–c) identified two groups of amplifications and deletions (Figure 5a,c): Cluster 1 represented isolates with a particular genetic background where SVs had high persistence times and low mutation counts; Cluster 2 isolates had SVs with low persistence times and elevated mutation counts. Amplifications had the highest persistence times (2.4 to 11.33% of the phylogeny) of all the SVs and were more frequent in the population (16.38 to 44.08 mutations towards amplified state; Figure S2). Therefore, we assessed the significance of differences in persistence times of amplifications between the two clusters (Figure 5d,e) and found that isolates with higher persistence times and low mutation counts were 16/20 times from common sources, whereas elevated mutation counts and low persistence times were associated with isolates from unusual hosts and host tissue (Table 1). These results suggest that isolates from uncommon sources may have more SVs, but that these mutations do not persist in the population. Isolates from common hosts have relatively few SVs, but these mutations are transmitted to extant lineages.

### 3.4. Stress-Related Environmental Conditions Drive SV Parallelism

Extensive studies of NA1 as an invasive pathogen have shown that forest conditions tightly control the survival and expansion of the population [19,24,29,30,31,32,76] and have the potential to drive the evolutionary trajectory. To identify geographic locations that may have isolates with SVs, plots were samples where NWT phenotype were previously identified (Figure 1b,c). NWT isolates are known to have SVs and are characterized by irregular growth in culture [10]. First, the NWT phenotype was tested for association with different types of SVs (amplification, translocation, deletion, all SVs, and parallel SVs, Table 2). The phenotypes of cultured *P. ramorum* NA1 isolates were scored as previously defined by Kasuga and co-workers (2016) [10]. The sample size for Monterey Co. was 92 isolates and 102 isolates for Sonoma Co. Thirty-six of the 102 isolates (35.29%) from Sonoma Co. and 15 of the 92 (14.71%) isolates from Monterey Co. showed the NWT phenotype. Since SV types are strongly correlated with each other, individual models were made for each type. We found that deletions (DELno.) had the highest positive relationship with NWT phenotype (Figure S5a) and in a model comparison (Table 1), these SVs consistently had Bayes Factors >10 that ranged from Strong to Decisive (Jeffreys, 1961). The results of this phenotype by genotype analysis suggest that accumulation of deletions or other SV mutations results in an NWT phenotype and not parallel SVs.

**Figure 6:**
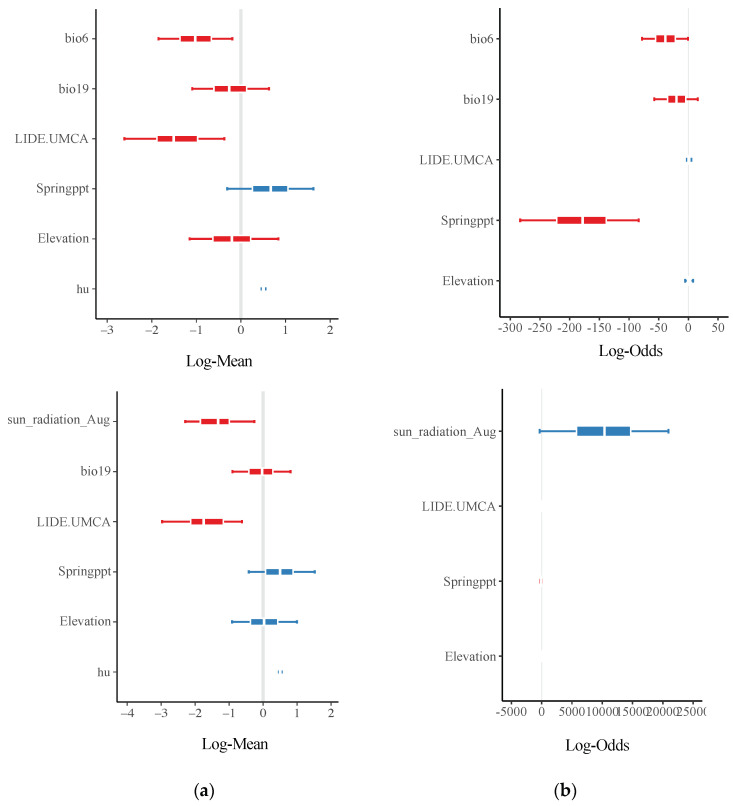
Correlation coefficients between forest environmental factors and genetic parallelism or phenotype. Posterior parameter estimates from Bayesian generalized linear hurdle Poisson models of parallel SVs in response to Jaccard’s distance of environmental factors (**a**). Posterior parameter estimates from a generalized linear model with a Bernoulli distribution (logit link function) of Jaccard’s distance between environmental conditions associated with NWT phenotype (**b**). Jaccard distance of environmental variables are calculated as the difference between Monterey Co. and Sonoma Co. First model (top panels) includes minimum temperature of the coldest month (bio 6), precipitation of the coldest quarter (bio19), *N. densiflorus* and *U. californica* stem density (LIDE.UMCA), precipitation during Spring (Springppt), Elevation, and a hurdle probability (hu). Second model (bottom panels) includes the same parameters, except solar radiation during August (sun radiation Aug) replaces minimum temperature during the coldest month. Bayes factor comparing both models was not significant (1.66).

Although NWT phenotype did not explain parallel SVs, we continued to ask if variables related to population expansion or survival could drive a repeated genetic response. We compared conditions that imposed stress on *P. ramorum* survival (e.g., solar radiation and minimum temperature during the coldest month) with variables involved in disease spread (e.g., spring and winter precipitation and elevation) to infer the environmental conditions associated with repeated evolution. First, a generalized linear model (GLM) framework in which environmental conditions were the independent variables and the number of parallel SVs were the response variables was constructed (Figure 6). All SVs except three translocations (excluded from all analyses) were parallel between Sonoma Co. and Monterey Co. sub-populations (Figure 6a). The coefficient estimates with credible intervals that did not traverse zero indicated greater confidence that these environmental parameters were drivers of parallelism. Moreover, Jaccard’s distance in minimum temperature during the coldest month was negatively correlated with the number of parallel SVs, thereby indicating that as conditions between counties become more similar, the number of parallel SVs increases.

Tree density of the main hosts, *N. densiflorus* and *U. californica*, also predicted the probability of genetic parallelism (Figure 6a). This factor was categorized under survival and transmission depending on the stage of pathogen life cycle and season. Since other conditions associated with population expansion were not explanatory variables of parallel SVs, our results suggest that conditions imposing stress on survival drive genetic parallelism. Jaccard’s distance for solar radiation was correlated with convergence in minimum temperature of the coldest month, so a second model with solar radiation and all other parameters except minimum temperature was tested. The correlation coefficient of similarity for solar radiation during the hottest month (“sun_radiation_Aug”) also indicated an association with parallel SVs. Bayes factor comparing both models was not significant (1.66), thus indicating that neither model performed significantly better than the other. Second, independent variables related to population expansion in the pathogen and survival under binomial models were also used to test for convergence of the NWT phenotype (Figure 6b). Similarity in spring precipitation predicted the probability of NWT isolates in both regions. Results suggest that the number of parallel SVs increased as environmental conditions involved in pathogen survival became more similar between Monterey Co. and Sonoma Co. (Table 1 and Figure 6a) and the generation of NWT isolates are driven by environmental variables involved in the pathogen’s population expansion (Figure 6b).

Model performance was validated using posterior predictive checks, and by inspection of the Markov chain trace plots. For all models, the observed response variable (“y”) was within the range of the simulated response variable (“y_rep_”). The estimates of the coefficients converged, as shown in the Markov chain trace plots (Figure S3) supporting model adequacy. Models associating genotype with phenotype were ranked according to their performance in comparison to all other models. Deletions showed the strongest correlation, while translocation and parallel SVs were the worst. Posterior predictive checks and Markov chain trace plots show that these models adequately predicted the proportion of NWT/NWT and NWT/WT or WT/WT pairs, and that the models had converged for the correlation coefficients (Figure S4).

### 3.5. SVs are Associated with Genes Involved in Pathogenesis

Given that new mutations can potentially alter pathogen behavior at the population level, we therefore tested if parallel SVs intersect with genic regions involved in pathogenesis. To this end, we counted base pair overlaps between parallel SVs and differentially up-regulated genes in *P. ramorum* associated with *N. densiflorus* infection were counted from an RNAseq analysis. Results of a Fisher-exact test showed that the odds of a parallel SV base pair overlapping with a base pair that was up-regulated in the pathogen during *N. densiflorus* infection was 1.15:1 (*p*-value < 2.2 × 10^−16^). Similarly, these SVs also overlapped with RXLRs (1.11:1), CRNs (1.95:1), and genic regions (1.12:1) greater than expected (*p*-value < 2.2 × 10^−16^) except for repetitive regions (odds ratio 0.60 and *p*-value < 2.2 × 10^−16^, Table 3).

Notably, the SV data set had a significant overlap with effector RXLRs (i.e., containing the amino acid motif arginine (R), any amino acid (X), leucine (L), and arginine (R) required for transport into the plant host cell [77,78]. Specifically, 27 amplification SV loci overlapped with 121 RXLRs, one of which also contained a translocation. The left-tailed test showed that the overlap between SVs and genomic features was greater than expected. The 16 SV loci with greater persistence times/mutation counts overlapped with 22 RXLRs, 43 WY domains, 234 repetitive regions, and 984 genic regions. These findings strongly suggest that SVs can potentially alter the pathogenic phenotype of *P. ramorum*.

Since parallel SVs can also potentially be associated with biological, molecular, or chemical functions in the pathogen, we performed a GO enrichment test of parallel SVs with longer persistence times against the whole genome of *P. ramorum* NA1 as background and found that pathogenesis, DNA binding/modification, motility, cell wall modification, and fertilization GO terms were over-represented among genes that overlap with parallel SVs (Supplementary File S2).

## 4. Discussion

With the combined analyses of genomic and environmental data, we were able to demonstrate another process of asexual evolution by somatic mutation and parallel evolution in the NA1 clonal lineage of *P. ramorum*. Parallel evolution at the genotypic level is defined as the independent evolution of homologous loci [79,80]. In addition, we have characterized the likelihood that parallel SVs persist in the population. Moreover, our results exemplified that these parallel SVs are likely a consequence of forest ecology and could interfere with biological function (e.g., pathogenicity, stress response, carbohydrate metabolism; Supplementary File S2) in isolates of *P. ramorum*.

Phylogeny confirmed SVs were somatic mutations and occurred on multiple lineages independently. After examining the linkage disequilibirum for all SNP markers, high levels of heterozygosity, and minimal discordance in a phylogenetic network there was no evidence for sexual recombination. Asexual reproduction in *Phytophthora ramorum* NA1 not only confirms that SVs are somatic mutations but that these mutations are undergoing an unusual process of parallel evolution. Genetic parallelism is an unusual phenomenon that draws the attention of evolutionary biologists and most studies indicate that these processes are usually not associated with genetic drift or chance mutations, but rather phenotypic evolution and selection from environmental parameters [81,82]. Our results showed that SVs transmitted to extant lineages and had the potential to change pathogenicity but, as of yet, we do not know the adaptive significance for this pattern in *P. ramorum* NA1. Future research examining SV’s association with pathogenicity-related phenotypes may reveal how the pathogen is adapting to its new range.

Parallel SVs are likely to be transmitted to extant lineages based on their genetic background. Our findings indicated that hosts unfamiliar, though potentially transmissive by supporting sporulation, to *P. ramorum* increased the generation of SVs and decreased their persistence (Table 2). Instead, isolates or lineages associated with common hosts (e.g., *U. californica* and *N. densiflorus*) had SVs which persisted longer in the population. Our estimates of persistence times could be affected by the population demographics. For example, at the forefront of a range expansion following founder effects, allele surfing could increase the frequency of certain alleles, including SVs, that would not have been retained by natural selection [83]. However, our examination of several demes that are undergoing parallel evolution supports that population demographics is not the main driver of longer persistence times of SVs. Whether SVs transmitted to extant lineages are therefore under positive selection requires further research.

Repeated evolutions of SVs arose in locations where environmental parameters that influence pathogen survival are converging. First, parallel SVs were associated with similarity in minimum temperature during the coldest month, host stem density, and solar radiation between Monterey Co. and Sonoma Co. This was reflected in previous phenotypic studies of the pathogen and *P. ramorum*’s disease cycle in California. *P. ramorum* growth and survival are severely affected by lower temperatures [84]. Second, forest composition, such a similarity in relative *U. californica* stem density and *N. densiflorus* stem density, determines survival and not just disease spread [19,24,31,32], and these factors were unsurprisingly correlated with parallel SVs. Third, UV radiation and sun exposure affect growth, survival, and sporulation [32,84]. Thus, convergence in solar radiation, temperature, and host density are likely possible drivers of parallel SVs.

Structural variants (SVs) found in the NA1 population may have altered the biology and ecology of the pathogen. Some regions of the genome affected by SVs have been shown to be upregulated when the pathogen infects *N. densiflorus* and such regions are generally involved in pathogenicity in many microbes (RXLRs [26], and carbohydrate metabolism [85]). Gene Ontology (GO) terms connected with motility (e.g., ciliary plasm and cell projection part) suggested that new mutations also affect spore motility. Oomycetes produce motile zoospores that have two flagella, which are involved in dispersal of the pathogen [86]. Oddly, fertilization terms were also identified with SVs. However, mutations associated with fertilization GO terms might indicate the loss of meiotic genes which corresponds with asexual reproduction in the *P. ramorum* NA1 population. Future research might be able to link repeated evolution and SVs to phenotypic change and adaptation related to pathogenicity, stress response, motility, and carbohydrate metabolism. The parallel SVs overlapping with RXLRs and genes upregulated during *N. densiflorus* infection would provide a starting point for subsequent investigation of individual genes that contribute to survival and pathogenicity.

The “non-wild type” (NWT) is the only easily scorable phenotype of *P. ramorum* NA1 and is associated with SVs. However, NWT was found to be most strongly correlated with the number of deletions, a type of SV, and not with parallel SVs. Dale and coworkers (2019) [22] found that loss of heterozygosity through deletions was prevalent in *P. ramorum* including in the NA1 lineage. We only identified one case out of four deletions classified as copy number neutral loss of heterozygosity (deletion at contig_74: 147088–149087 and amplification at contig_74_alt: 91020–148058) in our final dataset. Results suggested that at least for *P. ramorum* NA1, loss of heterozygosity arose in the population but did not persist long and did not likely transmit to future generations of the *P. ramorum* NA1 population. These results support Muller’s ratchet [87], the accumulation of slightly deleterious mutations, is likely driving the extinction of isolates or branches in the phylogeny that have elevated SV counts. Therefore, it is possible that deletions and NWT are a byproduct of a mechanism that generates SVs or new mutations in *P. ramorum* NA1. Perhaps other phenotypes (whether easily detected or not) are associated with parallel SVs. However, previous phenotypic studies have focused on variation between the four lineages (NA1, NA2, EU1, and EU2) or hardly detected phenotypic variation within the *P. ramorum* NA1 population [84,88,89].

## 5. Conclusions

By combining whole genome sequencing, phylogenetic inference, and ecological data, we were able to identify how genetic variation is generated in an asexual organism at the DNA level to the landscape level. *P. ramorum* NA1 was found to be strictly asexual and mutations arose somatically. Structural Variants were distributed on multiple independent lineages in locations where forest conditions are similar. Depending on their genetic background, these mutations are likely to shape *P. ramorum* NA1′s evolutionary trajectory. These mutations intersected with putative RXLRs and occurred in regions of the genome associated with pathogenesis, suggesting the potential for the emergence of new strains that could alter the course of the epidemic. Whether genetic parallelism and persistence of SVs are adaptive in their new environment requires additional research. Our findings provide a starting point for further hypothesis testing of environmental conditions shaping genomic architecture and phenotypic adaptation in *P. ramorum* NA1, and have broader implications for forest management.

## Figures and Tables

**Table 1:** Fisher’s Exact test comparing host association to SV persistence times and counts. Columns represent common and uncommon hosts (*U. californica*, *N. densiflorus*) and sources (twig, leaf, bark, stream water) of *P. ramorum*. Isolates with high persistence times and low mutation counts are in Cluster 1; isolates with low persistence times and high mutation counts are in Cluster 2. Odds ratio = 13.8 and *p*-value = 0.023.

	Host/Source	Common	Uncommon
Cluster			
1		16	4
2		1	4

**Table 2:** Posterior parameter estimates from generalized linear models (binomial distribution with a logit function) of NWT/WT phenotype in response to SV types and their counts (amplification/AMPno., translocation/BNDno., deletion/DELno., all SVs/SVno., and parallel SVs with higher persistence times/Parallel SIG_SV). Median values of coefficients for each county are shown and the 95% Credible Intervals. Bayes factors are calculated for each model relative to an alternative model.

Count by SV Type	Monterey Co.: Coef. Est.	Monterey Co.: 5%	Monterey Co.: 95%	Sonoma Co.: Coef. Est.	Sonoma Co.: 5%	Sonoma Co.: 95%	Bayes Factor Relative to Model/ AMPno.	Bayes Factor Relative to Model/ BNDno.	Bayes Factor Relative To Model/ DELno.	Bayes Factor Relative to Model/ SVno.	Bayes Factor Relative to Model/ Parallel SIG_SV
AMPno	0.08	−0.01	0.22	0.10	0.03	0.21	⏤	27.86	0.06	0.82	9.19 × 10^14^
BNDno	−0.39	−1.97	0.80	0.31	−0.48	1.47	0.04	⏤	0.00	0.03	3.10 × 10^13^
DELno	2.89	0.35	5.72	1.55	0.37	3.02	15.51	489.51	⏤	13.05	1.47 × 10^16^
SVno	0.07	−0.01	0.21	0.10	0.03	0.21	1.19	35.03	0.08	⏤	1.21 × 10^15^
Parallel SIG_SV	0.35	−0.25	1.08	0.35	−0.25	1.07	0.00	0.00	0.00	0.00	⏤

**Table 3:** Fisher’s exact test between parallel SVs with highest persistence times and genes up-regulated in *P. ramorum* NA1 when infecting *N. densiflorus*, pathogenicity genes (putative RXLRs and CRNs), genic regions, and repetitive regions. A contingency table was constructed showing the number of base pairs found in both the SV region and genomic feature (second column), unique to the genomic feature (third column), unique to the SV region (fourth column), and found in neither significant SV region nor genomic feature (fifth column). The odds ratio and *p*-value of the Fisher’s exact test is also shown in the sixth and seventh column, respectively.

Genomic Feature	Intersect SV and Genomic Feature	Unique to Genomic Feature	Unique to SV	Neither SV Nor Genomic Feature	Odds Ratio	*p*-Value
*N. densiflorus* RNAseq	58534	1095221	2635487	56523374	1.15	<2.2 × 10^−16^
RXLR	12691	243686	2681330	57374909	1.11	<2.2 × 10^−16^
CRN	1850	20319	2692171	57598276	1.95	<2.2 × 10^−16^
Genes	2200663	46065733	493358	11552862	1.12	<2.2 × 10^−16^
Repeats	459449	14743843	2234572	42874752	0.60	<2.2 × 10^−16^

## Supplementary Materials

The following are available online at https://www.mdpi.com/2076-2607/8/6/940/s1, Figure S1: Splitstree network of *Phytophthora ramorum* isolates based on allelic variation at six microsatellite loci, Figure S2: Persistence times vs. Mutations by SV type per loci, Figure S3: Posterior predictive distributions and MCMC traces, Figure S4: Correlation between SV type and phenotype, Table S1: List of isolates, year collected, host and host tissue, location, and sequencing information, Table S2: Microsatellite fragment lengths for all isolates included in the analyses, Table S3 SV length correlation with persistence times or number of mutations, Supplemental File S1 SV haplotype analysis, and Supplemental File S2 GO enrichment analysis.
