# Supplementary material for: Asexual Evolution and Forest Conditions Drive Genetic Parallelism in Phytophthora ramorum
Source: Microorganisms. 2020 Jun 22;8(6):940. doi: 10.3390/microorganisms8060940 (PMC7357085; doi:10.3390/microorganisms8060940)
Supplement: Supplementary file 1 [file microorganisms-08-00940-s001.zip › Supplementary Data/Supplementary_Figures.docx]

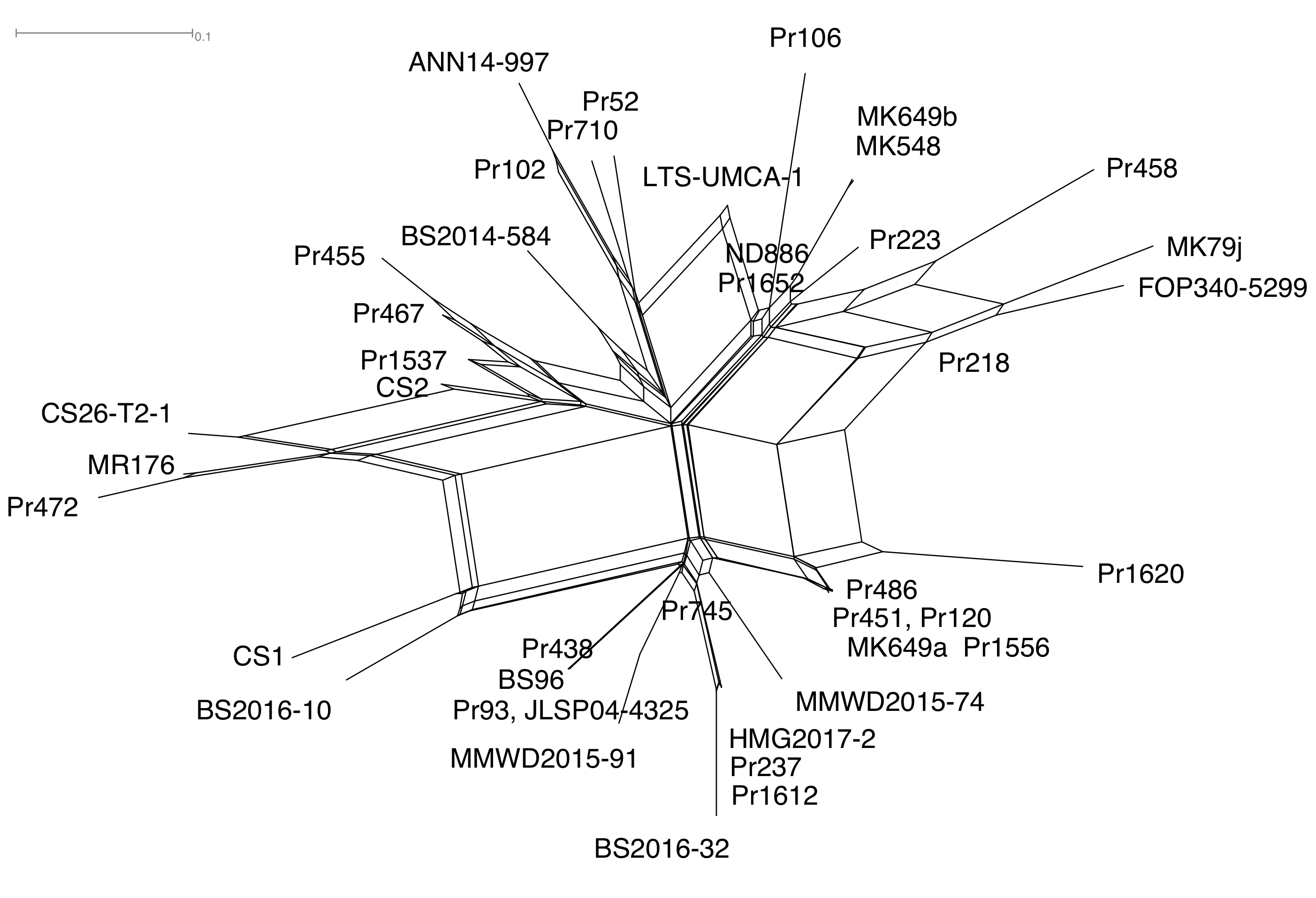


**Figure S1 Splitstree network of *Phytophthora ramorum* isolates based on allelic variation at 6 microsatellite loci**. Scale is Nei’s genetic distance (Da; Nei et al., 1983).

**Figure S2** **Persistence times vs Mutations by SV type per loci**. Persistence times are scaled to a percentage of the total time of the phylogeny on the y-axis and counts towards a mutation are on the x-axis. Each plot has 158 data. Kendall rank correlation was used to test for association between persistence times and mutations. Amplification type SVs (top left panel) have persistence times ranging from 2.40 to 11.33% and transitions to the amplified state ranging from 16.38 to 44.08. Translocations (top right panel) have slightly lower persistence times and mutation counts (0.28 to 7.15%, 11 to 30.03). Deletions (lower left panel) have the lowest persistence times and number of mutations (0.04 to 3.48%, 1.87 to 5.38). For each SV site, most of the population is spent in a normal suggesting an increase in persistence time of SVs in response to mutations towards an SV state. Only the normal persistence time vs number of mutations towards the normal state has a negative relationship (tau=-0.82, p-value 4.4x10-53) suggesting highly mutable regions tend to move to an SV state.

**Figure S3 Posterior predictive distributions and MCMC traces.** The above graphs illustrated the adequacy of Figure 6 models associating parallel SVs with environmental variables (a), and culture phenotypes with environmental factors (b). The observed distribution of the response variable (“y”, shown as a dark blue line) was within the posterior predictive distribution (“y_rep_”: light blue distribution) both Poisson hurdle and Bernoulli models. Trace plots are shown for estimates of environmental drivers: minimum temperature of the coldest month (bio6), precipitation of the coldest month (bio19), Stem density of tanoak and California bay laurel, Spring (March) precipitation (Springppt), Elevation, and solar radiation during August (sun radiation Aug).

**Figure S4 Correlation between SV type and phenotype**. (a) Estimates of SV type correlated with WT/NWT phenotype, (b) comparison of posterior predictive distribution of the binomial model to the observed number of WT/NWT phenotypes, and (c) trace plots. Estimates of correlation coefficients are shown for Monterey Co. and Sonoma Co. SV types are as listed: number of amplifications (AMPno.), translocations (BNDno.), deletions (DELno.), all SVs (SVno.), and parallel SVs (matches_sigSV).

**Table S1** List of isolates, year collected, host and host tissue, location, and sequencing information.

| **Isolate Identification** | **Year Collection** | **Host** | **Host Tissue** | **County** | **Collection Source** | **Coverage** | **Sequencing Center** | **Technical Control** | **Easting** | **Northing** |
| --- | --- | --- | --- | --- | --- | --- | --- | --- | --- | --- |
| 115-JROTH01-280-SD1 | 2006 | *Umbellularia californica* | leaf | Sonoma | N. Rank | 69.1 | UC Davis Genome Center | ND886Aug2018 Pr1556Aug2018 | 535957 | 4245360 |
| 137-MITSU03-6078-L1 | 2007 | *Umbellularia californica* | leaf | Sonoma | N. Rank | 70.2 | UC Davis Genome Center | ND886Aug2018 Pr1556Aug2018 | 537622 | 4242828 |
| 15-ARBIT04-303 | 2010 | *Umbellularia californica* | leaf | Sonoma | N. Rank | 91.6 | UC Davis Genome Center | ND886Aug2018 Pr1556Aug2018 | 540417 | 4241851 |
| 161-SUGAR13-271-L1 | 2007 | *Umbellularia californica* | leaf | Sonoma | N. Rank | 67.5 | UC Davis Genome Center | ND886Aug2018 Pr1556Aug2018 | 541187 | 4254925 |
| 17-ATHEA02-6925 | 2010 | *Umbellularia californica* | leaf | Sonoma | N. Rank | 69.9 | UC Davis Genome Center | ND886Aug2018 Pr1556Aug2018 | 540726 | 4243304 |
| 171-TERRI02-416-L1 | 2007 | *Umbellularia californica* | leaf | Sonoma | N. Rank | 69.1 | UC Davis Genome Center | ND886Aug2018 Pr1556Aug2018 | 535661 | 4256967 |
| 19-ELIOT01-2436 | 2010 | *Umbellularia californica* | leaf | Sonoma | N. Rank | 75.7 | UC Davis Genome Center | ND886Aug2018 Pr1556Aug2018 | 539162 | 4241404 |
| 21-FOP04-169 | 2010 | *Umbellularia californica* | leaf | Sonoma | N. Rank | 70.2 | UC Davis Genome Center | ND886Aug2018 Pr1556Aug2018 | 535934 | 4244657 |
| 22-FOP04-202 | 2010 | *Umbellularia californica* | leaf | Sonoma | N. Rank | 70.8 | UC Davis Genome Center | ND886Aug2018 Pr1556Aug2018 | 535934 | 4244657 |
| 32-JLSP05-4227 | 2010 | *Umbellularia californica* | leaf | Sonoma | N. Rank | 71.0 | UC Davis Genome Center | ND886Aug2018 Pr1556Aug2018 | 540335 | 4244058 |
| 35-JLSP08-4349 | 2010 | *Umbellularia californica* | leaf | Sonoma | N. Rank | 88.2 | UC Davis Genome Center | ND886Aug2018 Pr1556Aug2018 | 539632 | 4243555 |
| 45-JROTH05-282 | 2010 | *Umbellularia californica* | leaf | Sonoma | N. Rank | 64.6 | UC Davis Genome Center | ND886Aug2018 Pr1556Aug2018 | 535897 | 4243994 |
| 5-ANN15-843 | 2010 | *Umbellularia californica* | leaf | Sonoma | N. Rank | 69.1 | UC Davis Genome Center | ND886Aug2018 Pr1556Aug2018 | 533723 | 4253228 |
| 60-SDC03-4369 | 2010 | *Umbellularia californica* | leaf | Sonoma | N. Rank | 65.4 | UC Davis Genome Center | ND886Aug2018 Pr1556Aug2018 | 540931 | 4243866 |
| 62-SUGAR13-271 | 2010 | *Umbellularia californica* | leaf | Sonoma | N. Rank | 68.4 | UC Davis Genome Center | ND886Aug2018 Pr1556Aug2018 | 541187 | 4254925 |
| 8-ANN22-3037 | 2010 | *Umbellularia californica* | leaf | Sonoma | N. Rank | 86.6 | UC Davis Genome Center | ND886Aug2018 Pr1556Aug2018 | 533211 | 4251684 |
| 95-FOP333-207 | 2007 | *Umbellularia californica* | leaf | Sonoma | N. Rank | 71.5 | UC Davis Genome Center | ND886Aug2018 Pr1556Aug2018 | 535539 | 4243772 |
| 96-FOP340-179 | 2007 | *Umbellularia californica* | leaf | Sonoma | N. Rank | 70.3 | UC Davis Genome Center | ND886Aug2018 Pr1556Aug2018 | 535006 | 4243430 |
| ANN14-997 | 2007 | *Umbellularia californica* | leaf | Sonoma | N. Rank | 62.3 | UC Davis Genome Center | ND886Jul2017 Pr1556Jul2017 | 533258 | 4253127 |
| BS2014-125 | 2014 | *Notholithocarpus densiflorus* | twig | Monterey | D. Rizzo | 89.8 | UC Davis Genome Center | ND886Aug2018 Pr1556Aug2018 | 602030 | 4038131 |
| BS2014-236 | 2014 | *Notholithocarpus densiflorus* | twig | Monterey | D. Rizzo | 72.5 | UC Davis Genome Center | ND886Aug2018 Pr1556Aug2018 | 600964 | 4035905 |
| BS2014-267 | 2014 | *Umbellularia californica* | leaf | Monterey | D. Rizzo | 79.6 | UC Davis Genome Center | ND886Aug2018 Pr1556Aug2018 | 600964 | 4035905 |
| BS2014-268 | 2014 | *Umbellularia californica* | leaf | Monterey | D. Rizzo | 72.5 | UC Davis Genome Center | ND886Aug2018 Pr1556Aug2018 | 600964 | 4035905 |
| BS2014-30 | 2014 | *Umbellularia californica* | leaf | Monterey | D. Rizzo | 80.3 | UC Davis Genome Center | ND886Aug2018 Pr1556Aug2018 | 629493 | 3989433 |
| BS2014-408 | 2014 | *Notholithocarpus densiflorus* | twig | Monterey | D. Rizzo | 79.8 | UC Davis Genome Center | ND886Aug2018 Pr1556Aug2018 | 605735 | 4013894 |
| BS2014-531 | 2014 | *Notholithocarpus densiflorus* | twig | Monterey | D. Rizzo | 70.3 | UC Davis Genome Center | ND886Aug2018 Pr1556Aug2018 | 606896 | 4022563 |
| BS2014-535 | 2014 | *Umbellularia californica* | leaf | Monterey | D. Rizzo | 68.7 | UC Davis Genome Center | ND886Aug2018 Pr1556Aug2018 | 606896 | 4022563 |
| BS2014-550 | 2014 | *Umbellularia californica* | leaf | Monterey | D. Rizzo | 69.9 | UC Davis Genome Center | ND886Aug2018 Pr1556Aug2018 | 640726 | 3978472 |
| BS2014-570 | 2014 | *Umbellularia californica* | leaf | Monterey | D. Rizzo | 69.6 | UC Davis Genome Center | ND886Aug2018 Pr1556Aug2018 | 639355 | 3982390 |
| BS2014-584 | 2014 | *Notholithocarpus densiflorus* | bark | Monterey | D. Rizzo | 31.1 | UC Davis Genome Center | Pr1556Jun2015 | 641309 | 3972919 |
| BS2014-708 | 2014 | *Umbellularia californica* | leaf | Monterey | D. Rizzo | 70.8 | UC Davis Genome Center | ND886Aug2018 Pr1556Aug2018 | 625429 | 3994611 |
| BS2014-710 | 2014 | *Umbellularia californica* | leaf | Monterey | D. Rizzo | 68.4 | UC Davis Genome Center | ND886Aug2018 Pr1556Aug2018 | 625429 | 3994611 |
| BS2016-10 | 2016 | *Umbellularia californica* | leaf | Monterey | D. Rizzo | 69.2 | UC Davis Genome Center | ND886Jul2017 Pr1556Jul2017 | 625429 | 3994611 |
| BS2016-13 | 2016 | *Umbellularia californica* | leaf | Monterey | D. Rizzo | 70.8 | UC Davis Genome Center | ND886Aug2018 Pr1556Aug2018 | 626141 | 3994394 |
| BS2016-23 | 2016 | *Umbellularia californica* | leaf | Monterey | D. Rizzo | 91.6 | UC Davis Genome Center | ND886Aug2018 Pr1556Aug2018 | 628101 | 3992051 |
| BS2016-28 | 2016 | *Umbellularia californica* | leaf | Monterey | D. Rizzo | 69.9 | UC Davis Genome Center | ND886Aug2018 Pr1556Aug2018 | 605735 | 4013894 |
| BS2016-32 | 2016 | *Notholithocarpus densiflorus* | twig | Monterey | D. Rizzo | 91.8 | UC Davis Genome Center | ND886Aug2017 Pr1556Aug2017 | 602030 | 4038131 |
| BS2016-43 | 2016 | *Umbellularia californica* | leaf | Monterey | D. Rizzo | 78.6 | UC Davis Genome Center | ND886Aug2018 Pr1556Aug2018 | 629493 | 3989433 |
| BS2016-47 | 2016 | *Umbellularia californica* | leaf | Monterey | D. Rizzo | 82.8 | UC Davis Genome Center | ND886Aug2018 Pr1556Aug2018 | 640726 | 3978472 |
| BS96 | 2003/2004 | *Umbellularia californica* | leaf | Monterey | D. Huberli | 66.3 | UC Davis Genome Center | Pr1556unusual |  |  |
| CS1 | 2016 | *Notholithocarpus densiflorus* | leaf | San Mateo | C. Small | 114.1 | UC Davis Genome Center | ND886Aug2017 Pr1556Aug2017 | 561956 | 4139477 |
| CS2 | 2016 | *Umbellularia californica* | leaf | San Mateo | C. Small | 98.9 | UC Davis Genome Center | ND886Aug2017 Pr1556Aug2017 | 567650 | 4137672 |
| CS26-T2-1 | 2008 | *Umbellularia californica* | leaf | Sonoma | N. Rank | 60.4 | UC Davis Genome Center | ND886Jul2017 Pr1556Jul2017 | 510180 | 4248356 |
| FOP340-5299 | 2010 | *Umbellularia californica* | leaf | Sonoma | N. Rank | 121.6 | UC Davis Genome Center | ND886Aug2017 Pr1556Aug2017 | 535006 | 4243430 |
| HMG2017-2 | 2017 | *Umbellularia californica* | leaf | Santa Cruz | H. McCown | 73.6 | UC Davis Genome Center | ND886Jul2017 Pr1556Jul2017 | 580782 | 4103047 |
| JLSP04-4325 | 2010 | *Umbellularia californica* | leaf | Sonoma | N. Rank | 120.3 | UC Davis Genome Center | ND886Aug2017 Pr1556Aug2017 | 539803 | 4244462 |
| LTS-UMCA-1 | 2017 | *Umbellularia californica* | leaf | Santa Cruz | E. Bernhardt and T. Swiecki (Phytosphere Research) | 68.4 | UC Davis Genome Center | ND886Jul2017 Pr1556Jul2017 |  |  |
| MK548 | 2008 | *Umbellularia californica* | leaf | San Mateo | M. Garbelotto | 75.9 | UC Berekeley QB3 | MK548 |  |  |
| MK649a | 2008 | *Umbellularia californica* | leaf | San Mateo | M. Garbelotto | 34.9 | UC Davis Genome Center | Pr710 |  |  |
| MK649b | 2008 | *Umbellularia californica* | leaf | San Mateo | M. Garbelotto | 48.9 | UC Berekeley QB3 | MK548 |  |  |
| MK79j | 2008 | *Umbellularia californica* | leaf | San Mateo | M. Garbelotto | 34.4 | UC Davis Genome Center | Pr710 |  |  |
| MMWD2015-74 | 2015 | *Notholithocarpus densiflorus* | twig | Marin | H. Mehl | 71.4 | UC Davis Genome Center | ND886Jul2017 Pr1556Jul2017 | 532691 | 4197236 |
| MMWD2015-91 | 2015 | *Notholithocarpus densiflorus* | twig | Marin | H. Mehl | 67.5 | UC Davis Genome Center | ND886Jul2017 Pr1556Jul2017 | 526272 | 4206325 |
| MR176 | 2005 | *Umbellularia californica* | leaf | Marin | M. Garbelotto | 105.5 | UC Davis Genome Center | ND886Aug2017 Pr1556Aug2017 | 523431 | 4209352 |
| ND886Aug2017 | 2004 | *Camellia sp.* | leaf | Marin | C. Blomquist | 86.2 | UC Davis Genome Center | ND886Aug2017 Pr1556Aug2017 |  |  |
| ND886Aug2018 | 2004 | *Camellia sp.* | leaf | Marin | C. Blomquist | 71.0 | UC Davis Genome Center | ND886Aug2018 Pr1556Aug2018 |  |  |
| ND886Jul2017 | 2004 | *Camellia sp.* | leaf | Marin | C. Blomquist | 63.8 | UC Davis Genome Center | ND886Jul2017 Pr1556Jul2017 |  |  |
| ND886Jun2016 | 2004 | *Camellia sp.* | leaf | Marin | C. Blomquist | 55.2 | UC Davis Genome Center | ND886Jun2016 |  |  |
| Pr102 | 2001 | *Quercus* | bark | Marin | D. Rizzo | 66.6 | Sainsbury Laboratory | Pr745 |  |  |
| Pr106 | ~2001 | *Umbellularia californica* | leaf | Sonoma | D. Rizzo | 97.2 | UC Davis Genome Center | ND886Jun2016 |  |  |
| Pr120 | 2001 | *Notholithocarpus densiflorus* | unknown | Mendocino | D. Rizzo | 85.2 | UC Davis Genome Center | ND886Jul2017 Pr1556Jul2017 |  |  |
| Pr1537 | 2012 | *Abies grandis* | twig | Mendocino | D. Rizzo | 67.4 | UC Davis Genome Center | Pr1556unusual | 434564 | 4374416 |
| Pr1556Aug2017 | 2012 | *Umbellularia californica* | leaf | Santa Clara | T. Kasuga | 130.5 | UC Davis Genome Center | ND886Aug2017 Pr1556Aug2017 |  |  |
| Pr1556Aug2018 | 2012 | *Umbellularia californica* | leaf | Santa Clara | D. Rizzo | 70.6 | UC Davis Genome Center | ND886Aug2018 Pr1556Aug2018 |  |  |
| Pr1556Jul2017 | 2012 | *Umbellularia californica* | leaf | Santa Clara | T. Kasuga | 77.2 | UC Davis Genome Center | ND886Jul2017 Pr1556Jul2017 |  |  |
| Pr1556Jun2015 | 2012 | *Umbellularia californica* | leaf | Santa Clara | T. Kasuga | 50.5 | UC Davis Genome Center | Pr1556Jun2015 |  |  |
| Pr1556unusual | 2012 | *Umbellularia californica* | leaf | Santa Clara | T. Kasuga | 59.8 | UC Davis Genome Center | Pr1556unusual |  |  |
| Pr1612 | 2013 | *Notholithocarpus densiflorus* | twig | Mendocino | D. Rizzo | 145.3 | UC Davis Genome Center | ND886Aug2017 Pr1556Aug2017 | 468402 | 4339889 |
| Pr1620 | 2013 | *Notholithocarpus densiflorus* | twig | Sonoma | D. Rizzo | 80.8 | UC Davis Genome Center | ND886Jul2017 Pr1556Jul2017 |  |  |
| Pr1652 | 2014 | stream |  | Humboldt | D. Rizzo | 83.0 | UC Davis Genome Center | Pr1556unusual |  |  |
| Pr218 | 2002 | *Frangula californica* | unknown | Sonoma | D. Rizzo | 65.7 | UC Davis Genome Center | Pr1556unusual |  |  |
| Pr223 | ~2002 | *Umbellularia californica* | leaf | Humboldt | D. Rizzo | 119.3 | UC Davis Genome Center | ND886Aug2017 Pr1556Aug2017 |  |  |
| Pr237 | ~2002 | *Lysimachia latifolia* | unknown | Monterey | D. Rizzo | 123.7 | UC Davis Genome Center | ND886Aug2017 Pr1556Aug2017 |  |  |
| Pr438 | 2006 | *Arbutus menziesii* | stem | Mendocino | D. Rizzo | 71.8 | UC Davis Genome Center | Pr1556unusual |  |  |
| Pr451 | 2004 | *Sequoia sempervirens* | leaf | Alameda | D. Rizzo | 65.2 | UC Davis Genome Center | Pr1556unusual | 573985 | 4185434 |
| Pr455 | ~2005 | *Osmorhiza berteroi* | unknown | Sonoma | D. Rizzo | 59.1 | UC Davis Genome Center | Pr1556unusual |  |  |
| Pr458 | 2005 | *Adiantum jordanii* | leaf | Sonoma | D. Rizzo | 75.2 | UC Davis Genome Center | Pr1556unusual |  |  |
| Pr467 | 2006 | *Corylus cornuta* | leaf | Mendocino | D. Rizzo | 78.5 | UC Davis Genome Center | Pr1556unusual |  |  |
| Pr472 | 2006 | *Choisya ternata (ornamental, orange blossom)* | unknown | Marin | D. Rizzo | 70.6 | UC Davis Genome Center | Pr1556unusual |  |  |
| Pr486 | ~2006 | *Camellia sp.* | leaf | Yolo | D. Rizzo | 68.4 | UC Davis Genome Center | Pr1556unusual |  |  |
| Pr52 | ~2001 | *Rhododendron* | leaf | Santa Cruz | D. Rizzo | 91.7 | UC Davis Genome Center | ND886Aug2017 Pr1556Aug2017 |  |  |
| Pr710 | 2009 | *Umbellularia californica* | leaf | Santa Clara | K. Aram | 33.6 | UC Davis Genome Center | Pr710 |  |  |
| Pr745 | ~2010 | rainwater |  | Santa Clara | E. Bernhardt and T. Swiecki (Phytosphere Research) | 67.0 | Sainsbury Laboratory | Pr745 |  |  |
| Pr93 | ~2001 | *Rhododendron sp.* | leaf | Santa Cruz | D. Rizzo | 74.1 | UC Davis Genome Center | ND886Jul2017 Pr1556Jul2017 |  |  |

**Table S2 Microsatellite fragment lengths for all isolates included in the analyses**. Allele sizes are coded by 3-digit for each locus (in column) and 42 isolates (in row). Missing data are coded 0.

| Isolate No. | PrMS39a |  | PrMS39b |  | PrMS43a |  | PrMS43b |  | PrMS45 |  | locus64 |  |
| --- | --- | --- | --- | --- | --- | --- | --- | --- | --- | --- | --- | --- |
| Pr451 | 130 | 130 | 246 | 246 | 372 | 372 | 486 | 486 | 166 | 186 | 342 | 380 |
| Pr223 | 130 | 130 | 246 | 246 | 368 | 368 | 482 | 482 | 166 | 186 | 342 | 380 |
| Pr1652 | 130 | 130 | 246 | 246 | 368 | 368 | 0 | 0 | 166 | 186 | 342 | 380 |
| MMWD2015-74 | 130 | 130 | 246 | 246 | 372 | 372 | 494 | 494 | 166 | 186 | 342 | 380 |
| MMWD2015-91 | 130 | 130 | 246 | 246 | 372 | 372 | 490 | 490 | 0 | 0 | 342 | 380 |
| MR176 | 130 | 130 | 250 | 250 | 376 | 376 | 490 | 490 | 166 | 186 | 342 | 380 |
| ND886 | 130 | 130 | 246 | 246 | 368 | 368 | 0 | 0 | 0 | 0 | 342 | 380 |
| Pr102 | 130 | 130 | 246 | 246 | 364 | 364 | 490 | 490 | 166 | 186 | 342 | 380 |
| Pr472 | 130 | 136 | 250 | 250 | 376 | 376 | 490 | 490 | 166 | 186 | 342 | 380 |
| Pr120 | 130 | 130 | 246 | 246 | 372 | 372 | 486 | 486 | 166 | 186 | 342 | 380 |
| Pr438 | 130 | 130 | 246 | 246 | 372 | 372 | 474 | 474 | 166 | 186 | 342 | 380 |
| Pr467 | 130 | 130 | 246 | 246 | 376 | 376 | 482 | 482 | 166 | 186 | 342 | 380 |
| Pr1537 | 130 | 136 | 246 | 246 | 376 | 376 | 0 | 0 | 166 | 186 | 342 | 380 |
| Pr1612 | 130 | 130 | 246 | 246 | 372 | 372 | 482 | 482 | 166 | 186 | 342 | 380 |
| BS2014-584 | 130 | 130 | 246 | 246 | 378 | 378 | 0 | 0 | 0 | 0 | 342 | 380 |
| BS2016-10 | 130 | 130 | 250 | 250 | 372 | 372 | 482 | 482 | 166 | 186 | 342 | 380 |
| BS2016-32 | 130 | 130 | 246 | 246 | 372 | 372 | 482 | 482 | 156 | 186 | 342 | 380 |
| BS96 | 130 | 130 | 246 | 246 | 372 | 372 | 474 | 474 | 166 | 186 | 342 | 380 |
| Pr237 | 130 | 130 | 246 | 246 | 372 | 372 | 482 | 482 | 166 | 186 | 342 | 380 |
| CS-1 | 130 | 130 | 250 | 250 | 372 | 372 | 478 | 478 | 166 | 186 | 342 | 380 |
| CS-2 | 130 | 130 | 246 | 246 | 376 | 376 | 486 | 486 | 166 | 186 | 342 | 380 |
| MK548 | 130 | 130 | 246 | 246 | 368 | 368 | 494 | 494 | 166 | 186 | 342 | 380 |
| MK649a | 130 | 130 | 246 | 246 | 372 | 372 | 486 | 486 | 166 | 186 | 342 | 380 |
| MK649b | 130 | 130 | 246 | 246 | 368 | 368 | 494 | 494 | 166 | 186 | 342 | 380 |
| MK79j | 130 | 130 | 242 | 242 | 368 | 368 | 486 | 486 | 166 | 186 | 342 | 380 |
| Pr710 | 130 | 130 | 246 | 246 | 346 | 346 | 490 | 490 | 166 | 186 | 342 | 380 |
| Pr745 | 130 | 130 | 246 | 246 | 372 | 372 | 0 | 0 | 0 | 0 | 342 | 380 |
| Pr1556 | 130 | 130 | 246 | 246 | 372 | 372 | 486 | 486 | 166 | 186 | 342 | 380 |
| HMG2017-2 | 130 | 130 | 246 | 246 | 372 | 372 | 482 | 482 | 166 | 186 | 342 | 380 |
| LTS-UMCA-1 | 130 | 130 | 246 | 246 | 368 | 368 | 490 | 490 | 166 | 186 | 342 | 380 |
| Pr52 | 130 | 130 | 246 | 246 | 380 | 380 | 490 | 490 | 166 | 186 | 342 | 380 |
| Pr93 | 130 | 130 | 246 | 246 | 372 | 372 | 490 | 490 | 166 | 186 | 342 | 380 |
| ANN14-997 | 130 | 130 | 246 | 246 | 364 | 364 | 490 | 490 | 0 | 0 | 342 | 380 |
| CS26-T2-1 | 130 | 130 | 250 | 250 | 376 | 376 | 486 | 486 | 166 | 186 | 342 | 380 |
| FOP340-5299 | 130 | 130 | 250 | 250 | 368 | 368 | 486 | 486 | 166 | 186 | 342 | 380 |
| JLSP04-4325 | 130 | 130 | 246 | 246 | 372 | 372 | 490 | 490 | 166 | 186 | 342 | 380 |
| Pr106 | 128 | 130 | 246 | 246 | 368 | 368 | 478 | 478 | 166 | 186 | 342 | 380 |
| Pr218 | 130 | 130 | 246 | 246 | 368 | 368 | 486 | 486 | 166 | 186 | 342 | 380 |
| Pr455 | 130 | 130 | 246 | 246 | 376 | 376 | 482 | 482 | 166 | 166 | 342 | 380 |
| Pr458 | 130 | 130 | 244 | 244 | 368 | 368 | 0 | 0 | 186 | 186 | 342 | 380 |
| Pr1620 | 130 | 130 | 244 | 244 | 372 | 372 | 486 | 486 | 166 | 186 | 342 | 380 |
| Pr486 | 130 | 130 | 246 | 246 | 372 | 372 | 486 | 486 | 166 | 186 | 342 | 380 |

**Supplemental File 1 SV haplotype analysis**. Genomic ranges (contigs and positions) for SVs listed for haplotype 1 and 2. Datasheet “SVsHaps” indicate type of SV [amplification (“AMP”), translocation (“BND”), deletion (“DEL”), and no mutation (“Normal”)] are listed for each isolate. Datasheet “SVbothHaps” show SVs that occur in both haplotypes.

**Supplemental File 2 GO enrichment analysis**. GO terms associated with SVs with longer persistence times. Longer persistence times were calculated per branch (datasheets “sigSVsbranch_BP”, “sigSVsbranch_CC”, and “sigSVsbranch_MF”) and per loci (datasheets “sigSVsloci_BP”, “sigSVsloci_CC”, and “sigSVsloci_MF”). Datasheets appended with “BP”, “CC”, and “MF” indicate biological function, cellular component, and molecular function, respectively. The p-values were adjusted using a false discover rate as in Benjamini & Hochberg, 1995.
